# Usefulness of serum Mac-2 binding protein glycosylation isomer in patients undergoing hepatectomy: A case controlled study

**DOI:** 10.1016/j.amsu.2019.10.014

**Published:** 2019-10-17

**Authors:** Masahide Hiyoshi, Koichi Yano, Atsushi Nanashima, Makoto Ikenoue, Naoya Imamura, Yoshiro Fujii, Takeomi Hamada, Takahiro Nishida

**Affiliations:** a Division of Hepato-biliary-pancreatic Surgery and Department of Surgery, University of Miyazaki Faculty of Medicine, Miyazaki, Japan; b Division of Gastrointestinal, Endocrine and Pediatric Surgery, Department of Surgery, University of Miyazaki Faculty of Medicine, Miyazaki, Japan

**Keywords:** Mac-2 binding protein glycosylation isomer (M2BPGi), Hepatic fibrosis, Hepatectomy, Morbidity

## Abstract

**Background:**

To evaluate the clinical significance of Mac-2 binding protein glycosylation isomer (M2BPGi), we investigated the relationship between M2BPGi and clinicopathological and surgical parameters and posthepatectomy complications.

**Materials and methods:**

We examined M2BPGi in 115 patients with hepatic malignancies undergoing hepatectomy. Significance as an independent prognostic marker was determined with multivariate logistic regression analysis.

**Results:**

The mean serum M2BPGi level was 1.14 ± 1.03 C.O.I. (range 0.2–5.79). M2BPGi in the chronic viral hepatitis group (1.42 ± 1.25) was significantly higher than that in the other disease groups (p < 0.05). The M2BPGi level correlated negatively with platelet count, LHL15 and GSA-Rmax (r = −0.36, −0.69 and −0.56, respectively; p < 0.01) but correlated positively with serum hyaluronate level (fibrotic marker), ICGR15 and HH15 (r = 0.52, 0.63 and 0.57, respectively; p < 0.01). In 53 patients examined for histological hepatic fibrosis, the M2BPGi level was highest for hepatic fibrosis stage 4, indicating cirrhosis (2.15 ± 1.56), and was significantly higher than that for stages 0–2 (p < 0.05). M2BPGi level did not correlate significantly with any surgical parameters. The preoperative level correlated significantly only with increased alanine aminotransferase level (r = −0.21, p < 0.05) and was significantly higher in patients with (1.35 ± 0.78) than without (1.11 ± 1.07) hepatectomy-related complications (p < 0.05). Area under the ROC curve analysis for prediction of hepatic fibrosis score 4 showed a cut-off value of 0.78 for M2BPGi to have high sensitivity (90%) and specificity (58%). For postoperative hepatectomy-related complications, only the M2BPGi level (at a cut-off value 0.90) tended to show significance (p = 0.06).

**Conclusions:**

The non-invasively measured serum level of M2BPGi reflected impaired liver function or cirrhosis and hepatectomy-related complications after surgery, making it potentially useful as a complementary parameter accompanying other liver function parameters.

## Highlights

• The M2BPGi level was higher in patients with posthepatectomy complications.
• The M2BPGi level correlated negatively with the platelet count, LHL 15 and GSA-Rmax by liver scintigraphy.
• The M2BPGi level was highest in the patients with hepatic fibrosis.

## 1 Introduction

Hepatic resection is a useful radical treatment for various liver diseases [1,2], but background liver functional reserve or pathogenesis typically results in hepatic failure, uncontrolled ascites or surgical site infections [3,4]. Hepatic fibrosis or a severely injured liver can also cause severe postoperative complications, and precise preoperative evaluation of the presence of hepatic fibrosis is necessary to predict complications [5,6]. Precise preoperative evaluation of liver cirrhosis, which is the highest degree of hepatic fibrosis and a terminal situation, remains difficult. In place of invasive liver biopsy, examination of some serum markers of hepatic fibrosis and ultrasonic elastography have been applied to evaluate hepatic fibrosis [[7], [8], [9], [10]]. Some investigators including our group have reported that a serum marker such as hyaluronic acid level is a useful predictive marker for uncontrolled ascites or hepatic failure [11,12].

Mac-2 binding protein glycan isomer (M2BPGi), a novel marker for assessing hepatic fibrosis that was introduced over two decades ago, is a secreted glycoprotein present in the extracellular matrix of several organs [13] and induces inflammatory cytokines [14]. Human M2BP interacts with other extracellular collagens or fibronectin [15]. Bekki et al. reported that hepatic stellate cells are a source of M2BPGi [16], and M2BPGi levels reflect the activation of these cells during the process of liver fibrosis [17]. Furthermore, higher biological activities of M2BPGi are associated with the development of hepatocellular carcinoma [18]. Among the six candidate lectins binding M2BP [19], *Wisteria floribunda* agglutinin glycoprotein was identified as a M2BPGi. Some recent reports showed that M2BPGi was more closely associated with liver fibrosis than were other serum markers [20,21] and was a novel predictive biomarker for the responses to anti-viral hepatitis therapy [18,22], diagnosis of cirrhosis [20,21,23] and posthepatectomy liver failure in hepatocellular carcinoma (HCC) patients [24]. However, its clinical significance in the field of liver surgery has not yet been fully elucidated. We hypothesized that M2BPGi would be a novel predictive parameter for surgical outcomes, specific morbidity and histological findings in patients undergoing hepatectomy for various liver injuries. Our aim in this study was to clarify this aspect of the new predictive significance of this marker.

In the present study, we examined the serum values of M2BPGi in 115 patients with various liver diseases who underwent hepatectomy and considered the feasibility and limitations of this parameter as a supportive diagnostic modality in liver surgery.

## 2 Methods

### 2.1 Patients

In total, 115 patients with liver tumors admitted to the Division of Hepato-biliary-pancreatic Surgery at the University of Miyazaki Faculty of Medicine between September 2015 and March 2018 were consecutively examined. These liver tumors included HCC in 61 patients, intrahepatic cholangiocarcinoma in 5, colorectal liver metastasis in 27, extrahepatic bile duct carcinoma in 8, gall bladder carcinoma in 8 and other benign or malignant diseases in 6. The mean age of the 79 men and 36 women at the time of surgery was 66.6 ± 9.2 years (range, 24–85 years). The characteristics of the livers were normal liver in 33 patients, non-alcoholic steatohepatitis in 7, alcoholic in 2, chemotherapy-associated steatohepatitis (CASH) in 11, chronic viral liver injuries in 52 (including liver cirrhosis in 25) and obstructive jaundice in 10. The operative procedures included hemihepatectomy or more extended resection in 26 patients, segmentectomy or sectionectomy in 31 and partial resection in 58.

The study protocols were approved by the Human Ethics Review Board of our institution (approval no. and date: #O-0335, June 7, 2018). Agreement by the patients to enter the study was obtained by an opt-out procedure for one month at the website and outpatient clinic of our institution.

### 2.2 Measurement of serum M2BPGi

Peripheral blood samples were collected from each patient in the early morning before surgery, when the patient was in a stable condition. The blood sample was centrifuged at 3000 rpm for 15 min, and 0.4 mL of serum was stored at −80 °C until use. M2BPGi is measured using a chemiluminescent enzyme immunoassay with anti-WFA and anti-M2BP antibodies via a fully automated HSCL-2000i Immunoanalyzer (Sysmecs Co., Hyogo, Japan) [25]. The cut-off value was set at less than 1 cut-off index (C.O.I.) according to the company's data.

### 2.3 Clinicopathological parameters

The value of serum M2BPGi was compared in terms of patient demographics, conventional laboratory data, surgical records, histological findings and postoperative complications. Parameters of liver functional reserve tests that were compared included Child-Pugh classification, indocyanine green retention rate at 15 min (ICGR15), the parameters of ^99^m-technetium galactosyl serum albumin (GSA) liver scintigraphy (liver-uptake ratio between 3 and 15 min after injection [LHL15], blood pool clearance ratio between 3 and 15 min after injection [HH15] and the maximal removal rate of GSA [GSA-Rmax]) [26] and serum hyaluronic acid level [12]. Non-tumorous regions in the resected specimens that included liver tumors were used for assessment of histological findings, which were determined using Azan-Mallory and Elastica van Gieson stains by pathologists. Tumor staging and the grading score for hepatic fibrosis as defined by Knodell et al. were used for the histopathological evaluation [27].

### 2.4 Statistical analysis

All continuous data are expressed as mean ± SD. The data for the different liver disease groups were compared using one-way analysis of variance (ANOVA), which was examined by the Student *t*-test and the Scheffé’s multiple comparison test. The correlation of the continuous data was tested by Spearman's rank correlation test, and its correlation coefficient (r) is indicated. The sensitivity and specificity for each test value were calculated to assess the accuracy of scoring in differentiating between high and low degrees of fibrosis, and receiver operating characteristic (ROC) curves were constructed as the sensitivity against 1-specificity at each value. The index of accuracy was calculated by the area under the ROC curve (AUROC), in which a value close to 1.0 indicates high diagnostic accuracy. A two-tailed p value < 0.05 was considered significant. All statistical analyses were performed using the Statistical Package for the Social Sciences (SPSS) software, version 18.0 (IBM, Chicago, IL, USA).

## 3 Results

The mean serum M2BPGi level was 1.14 ± 1.03 C.O.I. and ranged between 0.2 and 5.79. Preoperative liver functions for each liver disease group are shown in Table 1 in comparison with those of the normal liver group. Most patients were classified as Child-Pugh grade A. Platelet count, LHL15 and GSA-Rmax were significantly lower in the patients in the chronic viral hepatitis group than in those in the other groups (p < 0.05). Meanwhile, ICGR15, HH15 and M2BPGi levels in the patients in the chronic viral hepatitis group were significantly higher than those in the other groups (p < 0.05). ICGR15 in the non-alcoholic, alcoholic, and CASH groups was also significantly higher than that in the normal liver group (p < 0.01). Total bilirubin level after biliary drainage in patients with biliary tumor obstruction was significantly higher than that in the other groups, but its range was less than 2.3 mg/mL.

**Table 1:** Liver function parameters in all 115 patients.

	Total (n = 115)	Normal liver (n = 33)	Non-alcoholic, alcoholic, CASH (n = 20)	Viral chronic hepatitis (n = 51)	Obstructive jaundice (n = 11)
Functional liver parameters					
Platelet count (10^4^/mL)	20 ± 8 (5–34)	24 ± 8 (15–43)	21 ± 7 (10–38)	15 ± 5 (6–28)*,**,***	23 ± 7 (14–39)
Prothrombin activity (%)	91 ± 17 (32–141)	95 ± 21 (79–127)	89 ± 18 (32–114)	89 ± 14 (63–141)	94 ± 17 (71–124)
Total bilirubin (mg/dL)	0.8 ± 0.3 (0.2–2.3)	0.7 ± 0.3 (0.3–1.5)	0.6 ± 0.3 (0.2–1.4)	0.8 ± 0.3 (0.3–1.6)	1.2 ± 0.6 (0.5–2.3)^¶^
HA (mg/dL)	102 ± 120 (4–780)	54 ± 45 (4–246)	116 ± 157 (9–566)	127 ± 139 (9–780)	102 ± 57(8–192)
ICGR15 (%)	11.8 ± 7.8 (1–50)	7.7 ± 4.5 (2–22)	14.3 ± 9.8 (2–40)*	14.1 ± 8.0 (1–34)^#^	8.7 ± 4.0 (4–15)
LHL15	0.93 ± 0.03 (0.79–0.97)	0.94 ± 0.02 (0.91–0.97)	0.93 ± 0.03 (0.88–0.97)	0.91 ± 0.04 (0.79–0.97)^#^	0.94 ± 0.02^†^ (0.90–0.97)
HH15	0.57 ± 0.09 (0.36–0.90)	0.53 ± 0.06 (0.43–0.66)	0.56 ± 0.08 (0.40–0.71)	0.60 ± 0.10 (0.36–0.90)^#^	0.53 ± 0.06 (0.39–0.63)
GSA-Rmax (mg/mL)	0.55 ± 0.17 (0.19–0.98)	0.61 ± 0.17 (0.23–0.98)	0.54 ± 0.18 (0.24–0.92)	0.50 ± 0.15 (0.19–0.78^§^	0.64 ± 0.17 (0.39–0.86)
M2BPGi (C.O.I.)	1.14 ± 1.03 (0.20–5.79)	0.64 ± 0.27 (0.24–1.32)	1.22 ± 1.17 (0.28–5.00)	1.42 ± 1.25 (0.20–5.79)*	1.20 ± 0.50 (0.40–1.89)
Child-Pugh classification					
(A/B)	114/1	33/0	20/0	51/1	11/0

Continuous data are shown as the mean ± SD with the range of minimum and maximum values in parentheses. Categorical data in parentheses are percentages.

*p < 0.01 vs. normal group, **p < 0.01 vs. obstructive jaundice group, ***p < 0.05 vs. non-alcoholic, alcoholic, CASH group, ¶p < 0.01 vs. other groups, #p < 0.01 vs. normal group, †p < 0.05 vs. viral chronic hepatitis group, §p < 0.05 vs. normal group.

CASH, chemotherapy-associated steatohepatitis; HA, hyaluronic acid level; ICGR15, indocyanine green retention rate at 15 min; LHL15, liver uptake ratio between 3 and 15 min, HH15, blood pool clearance ratio between 3 and 15 min of ^99m^-technetium GSA liver scintigraphy^35^; GSA, galactosyl serum albumin; M2BPGi (C.O.I.), mac-2 binding protein glycosylation isomer (cut-off index).

^#^Number of subjects was 113.

The relationship between the level of serum M2BPGi and the clinicopathological parameters is presented in Table 2. Age did not correlate with M2BPGi level, and there was no significant difference in this level between the sexes. For each background liver status, the patients with chronic viral liver injuries showed the highest M2BPGi level, which was significantly higher than that in the normal liver group (p < 0.01). In relation to the preoperative liver function parameters, the M2BPGi level correlated negatively with the platelet count, LHL15 and GSA-Rmax (p < 0.01) and correlated positively with the serum hyaluronate level (as a fibrotic marker), ICGR15 and HH15 (p < 0.01). However, the M2BPGi level did not correlate significantly with prothrombin activity or total bilirubin level.

**Table 2:** Relationship between serum M2BPGi and clinico-pathological parameters.

Age	r = 0.146
Sex	
Male (n = 78)	1.04 ± 0.80
Female (n = 37)	1.36 ± 1.39

Background liver disease	
Normal (n = 33)	0.64 ± 0.27
Non-alcoholic, alcoholic, CASH (n = 20)	1.22 ± 1.27
Chronic viral hepatitis or cirrhosis (n = 51)	1.42 ± 1.25^¶^
Obstructive jaundice (n = 11)	1.20 ± 0.50
Functional liver reserve	
Platelet count (/mm^3^)	r = −0.355**
Prothrombin activity (%)	r = −0.179
Total bilirubin (mg/dL)	r = 0.040
Serum hyaluronic acid level (mg/dL)	r = 0.516**
ICGR15 (%)	r = 0.628**
LHL15	r = −0.687**
HH15	r = 0.574**
GSA-Rmax (mg/mL)	r = −0.555**

Staging of hepatic fibrosis^a^	
0 (n = 4)	0.43 ± 0.20
1 (n = 8)	0.65 ± 0.32
2 (n = 12)	0.85 ± 0.68
3 (n = 12)	0.99 ± 0.46
4 (n = 17)	2.15 ± 1.56^#^

Surgical record	
Operation time (min)	r = −0.074
Transection time (min)	r = −0.183
Blood loss (mL)	r = 0.021

Postoperative liver functions^b^	
Total bilirubin level (mg/dL)	r = 0.021
Aspartate aminotransferase (IU/L)	r = −0.160
Alanine aminotransferase (IU/L)	r = −0.211*
Prothrombin activity (%)	r = −0.105
Platelet count (104/mm^3^)	r = −0.013
C-reactive protein (mg/dL)	r = −0.159

Postoperative hepatectomy related complications	
No (n = 98)	1.11 ± 1.07
Yes (n = 17)	1.35 ± 0.78*
Uncontrolled ascites	
No (n = 108)	1.13 ± 1.05
Yes (n = 7)	1.43 ± 0.87
Bile leakage	
Yes (n = 106)	1.11 ± 1.05
No (n = 9)	1.47 ± 0.87
Intra-abdominal infection	
No (n = 110)	1.15 ± 1.05
Yes (n = 5)	1.16 ± 0.85
Hepatic failure	
No (n = 113)	1.15 ± 1.05
Yes (n = 2)	1.14 ± 0.36

Hospital stay (days)	r = 0.033

Continuous data are shown as the mean ± SD. r, Pearson's correlation co-efficiency.

*p < 0.05, **p < 0.01, ¶p < 0.01 vs. normal liver group, #p < 0.05 vs. fibrosis scoring 0–2, respectively.

Abbreviations as in Table 1.

a Subjects were mainly examined for histological fibrosis according to Knodell's histological score [27] (n = 53).

b Maximum or minimum value within 7 days after hepatectomy.

In the 53 patients in whom histological hepatic fibrosis was examined, the M2BPGi level was the highest in the patients scored as hepatic fibrosis stage 4, indicating cirrhosis, and was significantly higher than that of patients with stages 0–2 (p < 0.05). The M2BPGi level was not significantly correlated with any surgical parameters. With respect to the changes in postoperative liver functions, the preoperative M2BPGi level was significantly correlated with an increase in the alanine aminotransferase level (p < 0.05) but otherwise was not correlated with other parameters of hepatic function. The preoperative M2BPGi level in patients with the presence of any hepatectomy-related complications was significantly higher than that in patients without complications (p < 0.05). However, there were no significant differences in M2BPGi level for each complication, such as ascites, bile leakage, infection and hepatic failure. The preoperative M2BPGi level was also not correlated with the length of hospital stay.

**Fig. 1:**
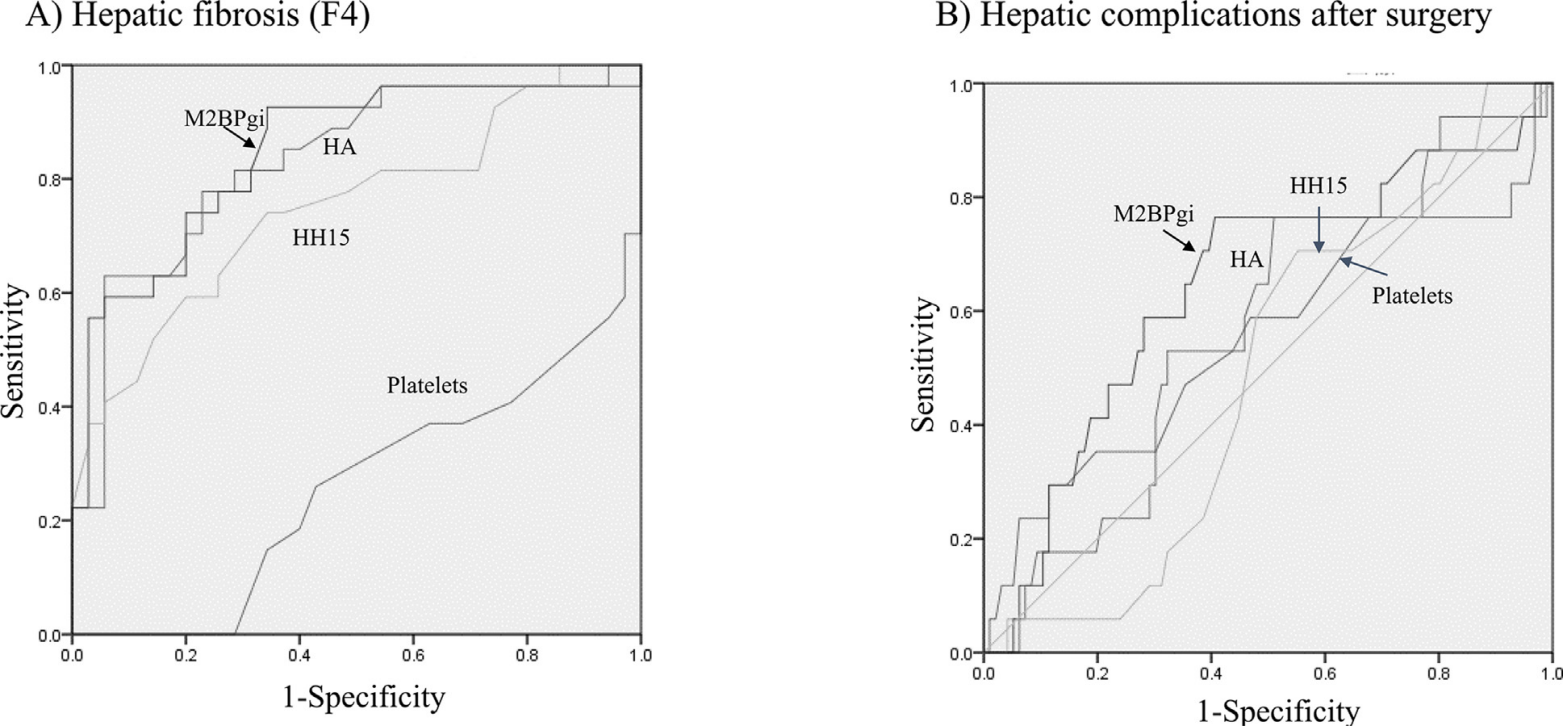
A) AUROC curve for stage 4 hepatic fibrosis, which indicates histological cirrhosis. Fibrotic markers and indexes such as serum hyaluronic acid (HA) level, platelet count, HH15 and M2BPGi were examined. B) AUROC curve for the existence of hepatic complications including hepatic failure, uncontrolled ascites, bile leakage and intra-abdominal infection. Fibrotic markers and indexes such as serum HA level, platelet count, HH15 and M2BPGi were similarly examined as in panel A.

The AUROC curves predicting stage 4 histological fibrosis, i.e., cirrhosis, for hyaluronate, platelet count, HH15 and M2BPGi are shown in Fig. 1A and Table 3. All four parameters showed significant associations (p < 0.01), and among them, M2BPGi at the cut-off level of 0.78 C.O.I. showed the highest sensitivity at 90% and a high specificity of 58%. With respect to the postoperative hepatectomy-related complications (Fig. 1B and Table 4), only the M2BPGi level tended to be significant (p = 0.057), and its cut-off value was set at 0.90 C.O.I.

**Table 3:** Area under receiver operating characteristics curve analysis between fibrotic markers and histological cirrhosis.

Parameters	Area	Standard deviation	Significance	95% Confidence interval		Cut-off value	Sensitivity (%)	Specificity (%)
				Lower limit	Upper limit			
HA	0.832	0.054	<0.01	0.727	0.937	130	63	62
Platelet count	0.257	0.064	<0.01	0.132	0.382	10	67	3
HH15	0.757	0.064	<0.01	0.626	0.876	0.60	74	34
M2BPGi	0.846	0.052	<0.01	0.948	0.948	0.78	90	58

Subjects were limited to HCC patients in whom liver fibrosis (F4) was examined histologically (n = 53).

Abbreviations as in Table 1.

**Table 4:** Area under receiver operating characteristics curve analysis between post-hepatectomy complications and fibrotic markers including M2BPGi.

Parameters	Area	Standard deviation	Significance	95% Confidence interval		Cut-off value
				Lower limit	Upper limit	
HA	0.575	0.072	0.327	0.433	0.717	70
Platelet count	0.551	0.087	0.500	0.380	0.722	19
HH15	0.490	0.065	0.898	0.362	0.610	0.60
M2BPGi	0.645	0.076	0.057	0.496	0.795	0.90

Abbreviations as in Table 1.

## 4 Discussion

The classic serum fibrotic markers and recent ultrasonography technology have allowed the precise measurement of liver fibrosis or impaired liver function [[29], [30], [31], [32]]. By applying these modalities, the diagnostic accuracy of liver fibrosis has been advanced without the necessity of performing invasive liver biopsies, and the cost of these examinations is relatively low. In recent years, as a more specific glycoprotein indicative of severe liver fibrosis or dysfunction, serum M2BPGi has been clinically applied and can be examined under national health insurance coverage in Japan. However, application of this marker has not been developed fully, particularly in the field of liver surgery, and only a few related reports have been published worldwide [21,24]. To further evaluate the clinical significance of the serum M2BPGi level to predict the surgical outcomes or morbidity, we thought evaluation in patients who have undergone hepatectomy to be necessary.

Serum M2BPGi serves as a fibrotic marker by detecting the alteration of glycoprotein caused by liver fibrosis as described above, which can be measured within 20 min using the presently available assay kit [20]. Hepatic stellate cells secrete M2BPGi, which may serve as a messenger between these cells and Kupffer cells via Mac-2 (galectin 3), which is expressed in Kupffer cells during the progression of fibrosis. So far, Nanashima et al. have reported the evaluation of hepatic fibrosis or liver stiffness by serum hyaluronic acid level or elastography [12,32]. According to these previous reports, the evaluation of preoperative liver fibrosis provided very useful information to predict postoperative uncontrolled ascites or hepatic failure when combined with other liver function parameters [12,24,33,34]. As the serum M2BPGi level is supposed to be more sensitive in reflecting hepatic fibrosis or liver damage according to recent reports, we hypothesized the additional significance of this new marker in the present study. Some reports revealed that the changes of M2BPGi before and after anti-viral hepatitis treatments reflected its high sensitivity in the evaluation of treatment effects [18,22,35,36].

In the present study, we examined several liver function parameters including M2BPGi in a background of various types of liver damage or liver disease. The M2BPGi level itself ranged widely between 0.2 and 5.8 C.O.I. although the cut-off value positive for liver cirrhosis was set at 1.0 C.O.I. according to the commercial data (in Japanese, http://www.falco.co.jp/business/047.pdf). Our present patient series was in the clinical setting of surgery, and therefore liver function was good, with most patients classified as Child-Pugh A. As expected, the chronic viral liver injury group, which included cirrhosis, showed the worst liver function data, and along with other parameters, the serum level of M2BPGi was also increased. However, in non-viral liver injuries such as non-alcoholic steatohepatitis, alcoholic injury or CASH, some of our patients showed a M2BPGi level as high as 4 or 5 C.O.I. The prevalence of liver cirrhosis in this group was lower in comparison to that of the viral hepatitis group. A M2BPGi level over 1.0 C.O.I. was observed even in the patients with a normal liver or obstructive jaundice due to biliary malignancies. The cut-off level to reflect any type of liver damage is wide-ranging [18,21,23], and a definitive cut-off value for cirrhosis has not been established yet. Therefore, the cut-off level indicated by the company data on the website listed above still needs to be investigated to reflect clinical significance in the present study.

By comparing clinicopathological parameters, surgical records and the post-hepatectomy outcomes shown in the present study, we found a correlation between M2BPGi and other conventional liver function parameters that has not been reported yet, to our knowledge. Decreased platelet counts and related parameters such as the Fibrosis-4 (F4) index or aspartate aminotransferase to platelet ratio index were closely associated with hepatic fibrosis [33,37,38]. Thus, a close correlation with decreased platelet counts might reflect the degree of liver stiffness. The serum hyaluronate level reflects not only hepatic fibrosis but also endothelial cell functions [12], which might better reflect impaired liver function when compared to type-IV collagen or other fibrotic markers. M2BPGi was closely correlated with serum hyaluronate level in the present results. ICGR15 is a standard liver function loading test used in Japan, and the parameters of GSA liver scintigraphy are often applied as a complementary liver function test [5,39]. M2BPGi correlated well with these loading tests in the present study, which also has not been previously reported. The basic strategies for indicating hepatectomy and estimating the resection rate are based on the results of ICGR15 at our institutions,^26^ and therefore, the range of ICGR15 is limited to within 35%. With respect to GSA liver scintigraphy, HH15 is thought to well reflect the impaired liver function in chronic liver injuries [40]. GSA-Rmax is also a parameter with which to predict posthepatectomy liver failure or uncontrolled ascites [41], but its independency as a predictive parameter might not be strong in comparison with ICGR15, as was shown by the multivariate analysis in our previous report [26]. In the present patient series, histological hepatic fibrosis was examined in only 53 patients with HCC or viral hepatitis, in whom the relationship between these scores and M2BPGi was assessed. M2BPGi was highly increased in F4 staging indicating cirrhosis. As shown by ROC analysis, the significance or sensitivity for histological fibrosis was the highest in comparison with the other related parameters in the present study. The cut-off value was set at approximately 0.8, which is lower than the commercial base cut-off value (1.0 C.O.I.) described above.

The degree of hepatic fibrosis might be associated with post-hepatectomy complications. Nanashima et al. reported that the value measured by ultrasonic elastography reflected intraoperative blood loss [32], and thus the relationship between M2BPGi and the surgical record was examined. However, no correlation was observed, contrary to our expectation. In comparison with the previous report by Nanashima et al. [33], the subjects of the present study were different and more recent; therefore, improvement of surgical techniques between the different study periods might have influenced this discrepancy. The preoperative M2BPGi level also did not reflect postoperative liver damage except for the level of alanine aminotransferase.

Prediction of posthepatectomy complications by considering the preoperative liver function parameters is necessary in the field of surgery. In the case of drug treatments for viral hepatitis, the M2BPGi level was useful in reflecting treatment responses [22,35]. Hepatic fibrosis might be closely associated with uncontrolled ascites and related liver failure as reported by Nanashima et al. [6,12,33]. Although the M2BPGi level was higher in the presence of total hepatectomy-related complications, the level was not significantly related to each individual complication in the present study. The ROC analysis showed the highest significance to be for hepatectomy-related complications in our series. In this situation, the cut-off value of 0.90 C.O.I. was slightly higher than that for reflecting hepatic fibrosis. Okuda et al. also reported the predictive value for hepatic failure in hepatectomy only for HCC, and their cut-off value ranged between 0.81 and 0.85 [24]. Therefore, the cut-off value for prediction of posthepatectomy complications might be set at approximately 0.8 or 0.9 C.O.I. based on the present results and those of Okuda et al. [24]. The preoperative M2BPGi level was also measured in the case of liver transplantation, and the value was much higher, over 2.0 C.O.I., than that of the present study [21]. In patients undergoing liver transplantation, the severity of cirrhosis or liver dysfunction level would be higher. To our knowledge, the present report might be the first to examine the significance of the M2BPGi level in various backgrounds of liver disease although the number of background subgroups was limited. It will still be necessary to examine the relationship between the M2BPGi level and endothelial cell function of the liver to elucidate the precise mechanism of this marker.

In conclusion, in this pilot study, we showed the potential for the newly developed serum fibrotic marker M2BPGi in patients with various backgrounds of liver disease who underwent various forms of hepatectomy. The serum M2BPGi level was higher in cirrhotic liver patients with F4 staging (i.e., cirrhosis), and it was significantly correlated with other reliable liver function parameters. The M2BPGi level was also higher in the patients with posthepatectomy complications. This non-invasive modality was very useful in the preoperative evaluation of chronic liver dysfunction and hepatic fibrosis, and therefore as the next step, it will be necessary to examine its utility in a larger number of patients to confirm its potential.

## Ethical approval

The study protocols were approved by the Human Ethics Review Board of our institution at the University of Miyazaki (approval no. and date: #O-0335, June 7, 2018).

## Sources of funding

No financial support was received for this study.

## Author contribution

Masahide Hiyoshi; Attending physician, writing the paper.

Koichi Yano; Attending physician, data collection.

Professor Atsushi Nanashima; director of our department who organised the study and checked the manuscript before submission.

Makoto Ikenoue; Attending physician, data collection.

Naoya Imamura; Attending physician, data collection.

Yoshiro Fujii; Attending physician, data collection.

Takeomi Hamada; Attending physician, data collection.

Takahiro Nishida; Attending physician, data collection.

## Research registration number

1. Name of the registry:

Usefulness of serum Mac-2 binding protein glycosylation isomer in patients undergoing hepatectomy.

2. Unique Identifying number or registration ID:

UMIN000036699.

3. Hyperlink to the registration (must be publicly accessible):

https://upload.umin.ac.jp/cgi-open-bin/ctr_e/ctr_view.cgi?recptno=R000041818.

## Guarantor

Atsushi Nanashima.

## Consent

Agreement by the patients to enter the study was obtained by an opt-out procedure for one month at the website and outpatient clinic of our institution.

## Disclosure

The authors declare that they have no conflicts of interest to disclose.

### Provenance and peer review

Not commissioned, externally peer reviewed.

## Declaration of competing interest

There are no conflicts of interest.
